# Borderline Personality Disorder With Cocaine Dependence: Impulsivity, Emotional Dysregulation and Amygdala Functional Connectivity

**DOI:** 10.3389/fpsyt.2018.00328

**Published:** 2018-07-31

**Authors:** Thania Balducci, Jorge J. González-Olvera, Diego Angeles-Valdez, Isabel Espinoza-Luna, Eduardo A. Garza-Villarreal

**Affiliations:** ^1^Clinical Research Division, National Institute of Psychiatry “Ramón de la Fuente Muñiz”, Mexico City, Mexico; ^2^Faculty of Medicine, National Autonomous University of Mexico, Mexico City, Mexico; ^3^Faculty of Psychology, National Autonomous University of Mexico, Mexico City, Mexico; ^4^Clinical Services Division, Psychiatric Hospital “Fray Bernardino Álvarez”, Mexico City, Mexico; ^5^Center of Functionally Integrative Neuroscience and MINDLab, Department of Clinical Medicine, Aarhus University, Aarhus, Denmark

**Keywords:** borderline personality disorder, cocaine dependence, dual pathology, functional connectivity, neuroimaging, amygdala

## Abstract

**Background:** Borderline personality disorder is present in 19% of cocaine dependence cases; however, this dual pathology is poorly understood. We wished to characterize the dual pathology and find its functional connectivity correlates to better understand it.

**Methods:** We recruited 69 participants divided into 4 groups: dual pathology (*n* = 20), cocaine dependence without borderline personality disorder (*n* = 19), borderline personality without cocaine dependence (*n* = 10) and healthy controls (*n* = 20). We used self-reported instruments to measure impulsivity and emotional dysregulation. We acquired resting state fMRI and performed seed-based analyses of the functional connectivity of bilateral amygdala.

**Results:** Borderline personality disorder and cocaine dependence as factors had opposing effects in impulsivity and emotional dysregulation, as well as on functional connectivity between left amygdala and medial prefrontal cortex. On the other hand, in the functional connectivity between right amygdala and left insula, the effect of having both disorders was instead additive, reducing functional connectivity strength. The significant functional connectivity clusters were correlated with impulsivity and emotional dysregulation.

**Conclusions:** In this study, we found that clinical scores of dual pathology patients were closer to those of borderline personality disorder without cocaine dependence than to those of cocaine dependence without borderline personality disorder, while amygdala-medial prefrontal cortex functional connectivity patterns in dual pathology patients were closer to healthy controls than expected.

## Introduction

Psychiatric comorbidities are present in 85–95% of cases of substance use disorders (1), and this entity is defined as dual pathology (2). Borderline personality disorder (BDP) with cocaine dependence (CD) as a dual pathology has been rarely studied, even though 19% of the patients with CD also have the BPD diagnosis (3). The dual pathology that includes cocaine dependence with borderline personality disorder will be written as BPD-CD from here on. BPD is characterized by a pattern of affect, self-image and interpersonal relationships instability, accompanied by impulsivity (4). Studies have found that CD is a consistent predictor for BPD with an odds ratio of 2.06, even higher than substances that have been more widely studied in relation to BPD such as alcohol or opiates (5, 6). Cocaine is a stimulant amine and it is the second most used illicit drug in Mexico, Central America, Western Europe and South Africa^1^ It has been estimated that 5–6% of those who consume cocaine develop dependence within the first year of use (7). This dependence is characterized by substance misuse with tolerance, abstinence syndrome, difficulties controlling consumption and clinical impairment or distress (4).

Women with BPD-CD show higher sexual risk behaviors compared to women with only CD and women with BPD and another substance use disorder (e.g., alcohol) (8). Men with BPD-CD show a greater attention bias to cocaine-related visual stimuli under emotional stress when compared to men with CD only (9). As impulsivity and emotional dysregulation are present in both conditions separately, these traits have been suggested as possible etiological factors of vulnerability to develop BPD-CD and may account for the between-group differences found (8–10). The results of these studies suggest a sex effect in patients with BPD-CD. Impulsivity can be defined as the tendency toward rapid unplanned reactions to internal or external stimuli without considering the consequences (11). Impulsivity increases risk for stimulant use disorder (12) and is a predictor for lifetime cocaine use (13). In BPD, impulsivity was the strongest predictor for borderline psychopathology over a 7-year follow-up (14). Emotional dysregulation is the difficulty to control and modulate one's affective state, such that emotions escape rational control and judgment (15). It is a core dimension of BPD and it is present during drug abstinence in CD patients (16).

Brain imaging studies have found differences in brain function measured by functional magnetic resonance imaging (fMRI) in both disorders separately (BPD and CD), when compared to healthy controls. In BPD, during an emotional processing task, amygdala activity was increased when exposed to fearful faces, while the anterior cingulate cortex (ACC) activity was decreased. The opposite activation pattern was shown for angry faces (17). Another study found hyperreactivity of amygdala, ACC and insula for negative and neutral pictures (18). Functional connectivity, defined as correlated remote neurophysiological events measured with fMRI (19), has been studied in these disorders during resting state. Studies have found higher functional connectivity between amygdala, insula and orbitofrontal cortex, and lower connectivity between ACC and posterior cingulate cortex (PCC). Studies in CD have found, lower connectivity between amygdala and medial prefrontal cortex (mPFC) and between ACC and posterior insula (20). Default mode network (DMN) connectivity, a network considered baseline for brain activity and related to autobiographical memory, seems to also be disrupted in both disorders (BPD and CD), with higher functional connectivity between mPFC and precuneus/PCC in BPD (21), and higher connectivity in ventromedial prefrontal cortex, precuneus and PCC has been found in CD (22). Amygdala, mPFC, ACC, PCC, precuneus and insula are brain regions involved in emotional regulation (23–27). However, the role of impulsivity and emotional dysregulation in BPD-CD are yet unknown, and there are no published functional connectivity studies addressing this dual pathology.

We aimed to investigate impulsivity, emotional dysregulation and functional connectivity, measured through fMRI in BPD-CD compared to the single pathologies and healthy controls. We hypothesized an additive effect both in the clinical domain. Regarding functional connectivity, according to the literature we expected increased amygdala functional connectivity in patients with BPD, which would be decreased in patients with CD, and a counteracting effect in the BPD-CD.

## Materials and methods

### Participants

The sample consisted of 69 participants divided into four groups: 20 with dual pathology of cocaine dependence and borderline personality disorder (BPD+CD+), 19 with cocaine dependence without borderline personality disorder (BPD−CD+), 10 with borderline personality disorder without cocaine dependence (BPD+CD−) and 20 controls without psychopathology (BPD−CD−). Demographic characteristics of the sample are summarized in Table 1. Participants were recruited from the outpatient Addiction Clinic and the Borderline Personality Disorder Clinic at the Instituto Nacional de Psiquiatría “Ramón de la Fuente Muñiz” and from the Xochimilco Toxicological Medical Unit (substance abuse treatment clinic) in Mexico City. The patients were a subsample from an ongoing cocaine addiction study (28). The BPD-CD- group was recruited via flyer advertisements and word of mouth. The groups were matched for sex, age, handedness and economic status. All participants provided written informed consent. The study followed the guidelines outlined in the Declaration of Helsinki and was approved by the Ethics Committee of the Instituto Nacional de Psiquiatría “Ramón de la Fuente Muñiz”.

**Table 1:** Demographic characteristics of the study participants.

	**BPD+CD+ (*n* = 20)**	**BPD−CD+ (*n* = 19)**	**BPD+CD− (*n* = 10)**	**BPD−CD− (n = 20)**	***F/X*^2^ value**	***p*-value**
Age, years (*SD*)	31 (7)	31 (6)	31 (13)	32 (8)	0.173	0.914
Gender: male, *n* (%)	15 (75)	18 (94)	5 (50)	18 (90)	10.16	<0.05
Education, median	High school	Junior high school	Technical degree	Technical degree	11.98	<0.01
Economic status^*^, median	D+	C	C/C+	C	4.071	0.254
Employment, *n* (%)						
Full-time	7 (35.0)	8 (42.1)	2 (20.0)	10 (50.0)	22.27	0.220
Half-time, formal	2 (10.0)	4 (21.1)	2 (20.0)	2 (10.0)		
Half-time, informal	5 (25.0)	5 (26.3)	2 (20.0)	2 (10.0)		
Student	1 (5.0)	2 (10.5)	4 (40.0)	4 (20.0)		
Housekeeper	–	–	–	1 (5.0)		
Home	2 (10.0)	–	–	–		
Unemployed	3 (15.0)	–	–	1 (5.0)		
Marital status, *n* (%)						
Unmarried	7 (35.0)	8 (42.1)	6 (60.0)	10 (50.0)	3.331	0.766
With partner	6 (30.0)	6 (31.6)	2 (20.0)	7 (35.0)		
Divorced/separated	7 (35.0)	5 (26.3)	2 (20.0)	3 (15.0)		
Laterality: right-handed, *n* (%)	19 (95.0)	14 (73.7)	10 (100)	17 (85.0)	6.392	0.381

* *The instrument used was the AMAI rule 8x7 created for Mexican homes, where A/B is the highest economic status category and E is the lowest*.

*BPD, borderline personality disorder; CD, cocaine dependence*.

For BPD screening, we used the self-report version of the Structured Clinical Interview for the Diagnostic and Statistical Manual of Mental Disorders 4th edition Axis II and the diagnosis was made with the Diagnostic Interview for Borderline Revised administered by a psychiatrist trained on personality disorders. Cocaine dependence was diagnosed using the MINI International Neuropsychiatric Interview Spanish version which was administered by two attending psychiatrists and two third-year psychiatry residents who were supervised by the attending psychiatrists.

The MINI International Neuropsychiatric Interview was also used to diagnose psychiatric comorbidity. Participants with bipolar, psychotic, obsessive-compulsive and eating disorders were excluded. For the BPD+CD- and BPD-CD- groups, the presence of any substance abuse or dependence except nicotine was an exclusion criterion. BPD+CD+ and BPD-CD+ groups could have another substance use disorder if cocaine was the primary substance of abuse. Cocaine consumption had to be active or with abstinence less than 60 days prior to the scan, with frequency of use of at least 3 days per week and no more than 60 continued days of abstinence during the last 12 months. Additional exclusion criteria for all groups were: somatic diseases (including neurological disorders), severe suicidal risk, history of head trauma with loss of consciousness, pregnancy, obesity, and noncompliance with magnetic resonance imaging safety standards. BDP-CD- participants presenting any psychiatric or somatic disorder were excluded.

### Clinical measures

Self-reported impulsivity was evaluated with the Barratt Impulsiveness Scale (BIS-11) which has three subscales: non-planning impulsiveness, which involves a lack of forethought; cognitive impulsivity, which involves making quick decisions; and motor impulsivity, which involves acting without thinking (29). Emotional dysregulation was assessed with the Difficulties in Emotion Regulation Scale (DERS) validated in Mexico (30), which, unlike the original version, it has 24 items and five subscales: non-acceptance of emotional responses, difficulty engaging in goal-directed behavior, lack of emotional awareness and lack of emotional clarity. Severity of CD was assessed using the Addiction Severity Index (31) Spanish version. For the CD+ groups, craving at the time of the MRI acquisition was evaluated with the Cocaine Craving Questionnaire—Now (32) in Spanish. The severity of BPD was assessed using the Clinical Global Impression Scale for BPD.

### Magnetic resonance imaging acquisition

Imaging data were obtained using a 3.0 Tesla Philips Ingenia magnetic resonance imaging scanner with a 32-channel phased array head coil. For the resting state fMRI, participants were instructed to remain quiet, relaxed and presented with cross. T2^*^-weighted echo planar images were acquired for 10 min (300 axial slices, repetition time = 2,000 ms, echo time = 30 ms, flip angle = 75°, field of view = 240 mm, slice thickness = 3.0 mm, acquisition matrix = 80 × 80 and voxel size = 3.0 × 3.0 × 3.0 mm^3^). Before this sequence, we acquired a field map correction sequence with the opposite acquisition direction. Then we acquired a T1-weighted sequence (repetition time = 7 ms, echo time = 3.5 ms, flip angle = 8°, field of view = 240 mm, slice thickness = 1.0 mm, acquisition matrix = 240 × 240 and voxel size = 1.0 × 1.0 × 1.0 mm^3^). As part of the main ongoing project, diffusion tensor imaging and fast diffusion kurtosis imaging sequences were also acquired with their field map correction and were not used in this study. Headphones were used to minimize noise exposure and to allow communication, and an eye tracker camera was used to ensure participants remained awake during the resting state fMRI sequence.

### Demographic and clinical statistical analysis

Demographic and clinical measures were compared with chi-square tests for categorical variables and Kruskal-Wallis test was used for ordinal variables. Significant between-group differences were followed by pairwise Mann-Whitney U tests with *p* < 0.01 using the Bonferroni correction for multiple comparisons. For continuous variables, a factorial two-way ANOVA was performed if criteria were met, with CD (+/−) and BPD (+/−) as factors. Post-hoc one-way ANOVAs with Tukey correction for multiple comparisons was used to assess between-group differences. To ensure a proper inference model, the analyses were repeated as ANCOVAs introducing demographic and comorbidity variables as confounds in the models, introducing each in separate models. If after removing outliers and normalizing variables, criteria for ANOVA analysis were not met, we used non-parametric analysis (Kruskal-Wallis and Mann-Whitney U). For clinical scales with <20% missing values, multiple imputation was performed using the automatic method from IBM SPSS 22.0, which performs a monotonic or conditional specified method depending upon de pattern of missing values. If there were more than 20% missing values, the participant was eliminated. Analyses were carried out using IBM SPSS 22.0. (IBM Corp. Released 2013. IBM SPSS Statistics for Windows, Version 22.0. Armonk, NY: IBM Corp).

### Magnetic resonance imaging processing and analysis

T1w images were preprocessed using an in-house pipeline with the software Bpipe (http://cobralab.ca/software/minc-bpipe-library/) (33), which uses the MINC Tool-Kit (http://www.bic.mni.mcgill.ca/ServicesSoftware/ServicesSoftwareMincToolKit) and ANTs (34). Briefly, we performed N4 bias field correction (35), linear registration to MNI-space using ANTs, cropped the region around the neck to improve registration quality, followed by transformation back to native space and brain mask creation. The fMRI images were preprocessed and analyzed using FSL 5.0.8 (36) and AFNI (37). Preprocessing included: slice-timing correction, motion correction, field map correction with the FieldMap Topup tool with opposite acquisition direction, brain extraction, segmentation, extraction of the global signal, cerebrospinal fluid signal, white matter signal, physiological noise reduction using aCompCor with 5 principal component analysis factors 38, coregistration, normalization to Montreal Neurological Institute (MNI) stereotactic space, bandpass filtering at 0.01 - 0.08 Hz and smoothing with a 6 mm Gaussian kernel.

We performed seed-based analyses using three seeds: right and left amygdala (lAmy/rAmy) consisting in 3 mm^3^ spheres which were created using fsl-maths and the MNI coordinates ± 26, 0, 20 based on previous localization of the amygdala (39). The third seed was for the default mode network (DMN), created using the areas: mPFC, PCC, medial temporal cortex and rostrolateral prefrontal cortex, from the Harvard-Oxford atlas (40). Then, we extracted the correlation coefficients between each seed and whole brain using FSL for our four regions of interest (ROIs), which were: mPFC, ACC, right and left insula (rIns/lIns), and DMN regions. The masks for the ROIs were taken from the Harvard-Oxford atlas. The second level analysis was done using a two-way ANOVA with CD (+/−) and BPD (+/−) as factors, constrained by five ROIs: bilateral mPFC, ACC and bilateral Insula from Harvard-Oxford atlas. For the main effects and interactions, we performed an *F*-test with FSL randomize (5,000 permutations) controlling for sex, age, education, current major depressive disorder, current dysthymia and current alcohol use, and followed by pair-wise *post-hoc T*-tests. For multiple comparison correction we used the family-wise error (FWE) at 0.05. Finally, as post-hoc analyses, Pearson's correlations were performed between the average correlation scores within significant clusters and scores from the BIS-11 and DERS questionnaires.

## Results

### Demographic data

Even with our efforts in recruitment, the groups differed significantly in sex and education due to the differences in sample sizes between groups. For this reason, sex and education were included as confounding variables in the inference models. Demographics are summarized in Table 1.

### Clinical findings

The psychiatric comorbidity and medications of the clinical groups are summarized in Supplementary Material Table 1S. We found significant differences on current major depressive disorder, current dysthymia, current alcohol use, number of cigarettes consumed per day and antidepressant use. These variables were also included as confounding variables in the analysis of clinical data. In terms of cocaine consumption, in the CD+ groups, we found no difference in age of onset (BPD+,CD+ M = 21.0 years, *SD* = 6.3; BPD−,CD+ M = 22.0, *SD* = 6.18; *U* = 160.5, *p* = 0.563), years consuming (BPD+,CD+ M = 7.4 years, *SD* = 5.4; BPD−,CD+ M = 8.6, *SD* = 6.18; *U* = 163.5, *p* = 0.624), administration route (smoked: BPD+,CD+ *n* = 13, 68.4%; BPD−,CD+ *n* = 11, 57.9%; *X*^2^ = 0.11, *p* = 0.737), amount of money spent on cocaine during the last 30 days (BPD+,CD+ M = 149.36 USD, *SD* = 342.66; BPD−,CD+ M = 185.11, *SD* = 203.90; *U* = 143.0, *p* = 0.180; exchange rate of Mexican pesos to USD at November 7, 2017: 19.14), presence of cocaine in urine (positive: BPD+,CD+ *n* = 6, 40.0%; BPD−,CD+ *n* = 7, 53.8%; *X*^2^ = 0.537, *p* = 0.724), craving (BPD+,CD+ M = 140.0 points on Cocaine Craving Questionnaire-Now, *SD* = 37.7; BPD−,CD+ M = 138.0, *SD* = 54.0; *F* = 0.30, *p* = 0.863), and addiction to cocaine severity (BPD+,CD+ M = 33.6 points on the Addiction Severity Index, *SD* = 15.9; BPD−,CD+ M = 26.3, *SD* = 17.8; *F* = 1.848, *p* = 0.182). The BPD groups did not differ in severity of BPD (Clinical Global Impression-BPD: BPD+, CD+ M = 3.61 points, *SD* = 1.30; BPD+, CD− M = 3.90, *SD* = 0.92; *F* = 0.394, *p* = 0.535).

**Figure 1:**
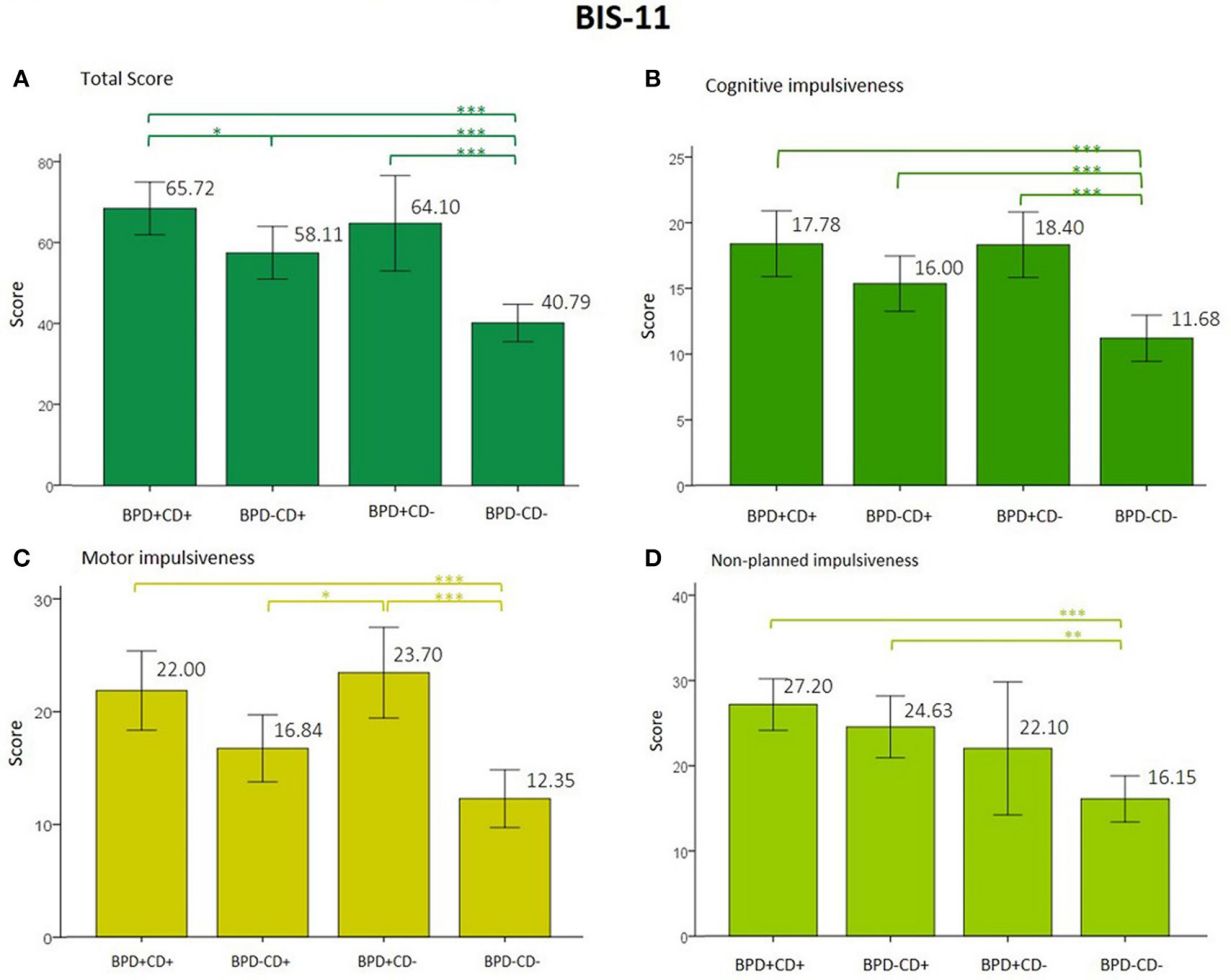
**(A–D)** Score from the total and subscales from the BIS-11. *p* value was corrected for multiple comparisons to < 0.01. Note: ^*^p < 0.05, ^**^p < 0.01, ^***^p < 0.001. BIS-11, Barratt Impulsiveness Scale; BPD, borderline personality disorder; CD, cocaine dependence.

Results of impulsivity are shown in Figure 1. Clinical groups showed lower impulsivity scores compared to controls without psychopathology. In the BIS-11 total score analysis, the BPD and CD main factors and interaction were significant. This interaction shows opposite effects, meaning that cocaine dependency showed lower BIS-11 total scores than borderline personality. However, the ANCOVA was only significant with some confounding variables. The use of antidepressants and daily consumption of cigarettes were added to the model, BPD and CD factors remained significant, but the interaction did not. The best fitting model was obtained with daily consumption of cigarettes as a confounding variable, accounting for 48.9% of the variance. For cognitive and motor impulsivity of the BIS-11, the BPD factor was significant and the best fit for the model was obtained when daily consumption of cigarettes was included, accounting for 41.4% and 36.1% of the variance, respectively. For non-planning impulsivity, also the main factor of CD was significant. The analyses and results are shown in Supplementary Table 2S.

**Figure 2:**
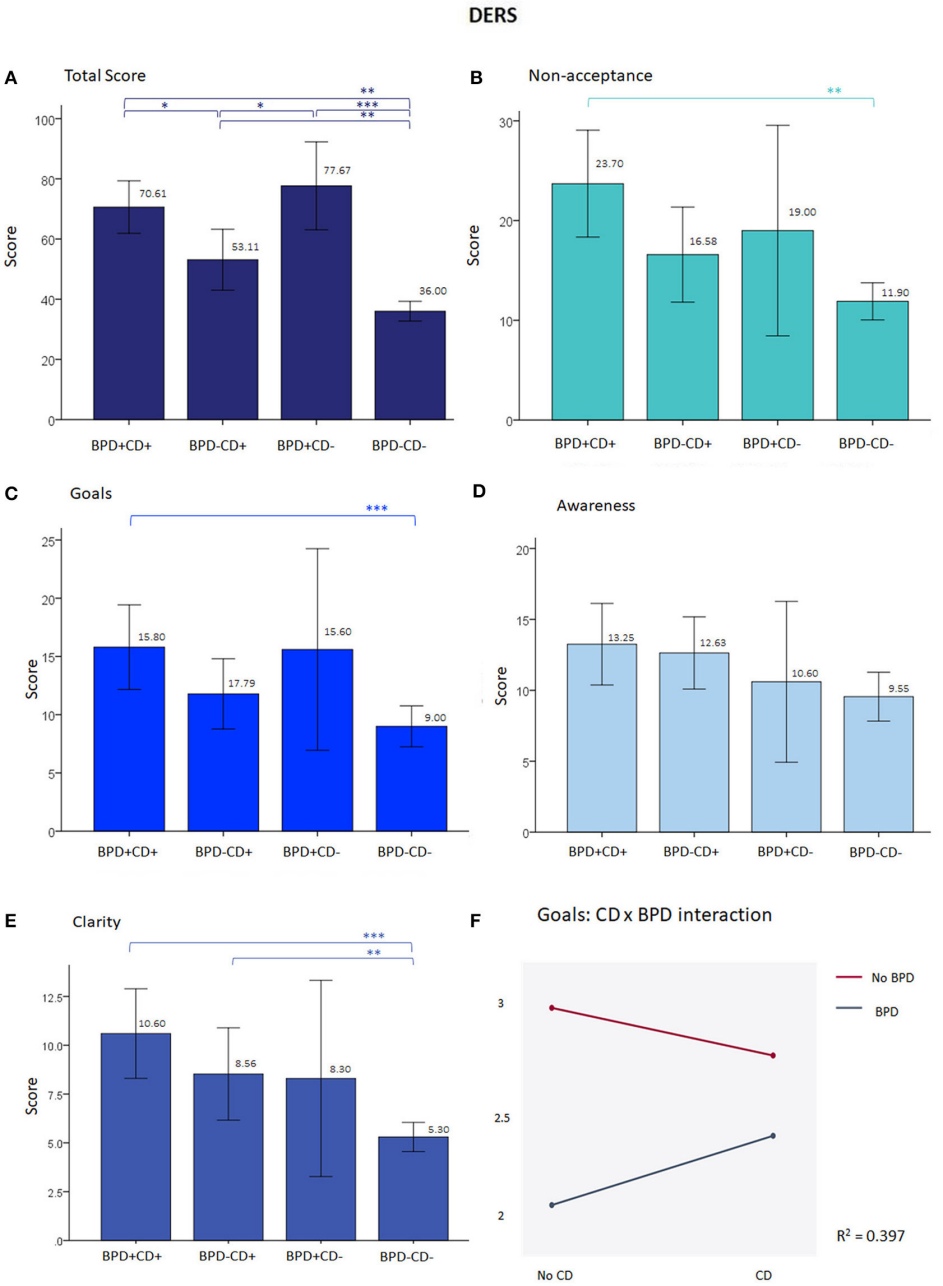
Results from the DERS. **(A)** At total scores, besides the difference between each clinical group and the BPD−CD− group, the difference is near significance between the BPD-CD+ and the BPD+CD+ groups, and the former with the BPD+CD−. **(B–C)** show graphs with a similar shape than **(A)**, but without the significant results. **(D)** There were no differences between groups in awareness subscale. **(E)** For clarity subscale only CD groups differed from BPD-CD-. **(F)** Negative significant interaction from the ANOVA at goals subscale (*F* = 6.19, *p* = 0.05) and in the borderline personality disorder factor (*F* = 34.84, *p* < 0.001). When adding cigarettes/day as covariate, the *R*^2^ improves to 0.421, remaining the interaction significant (*F* = 5.33, *p* < 0.05) and the BPD factor (*F* = 32.04, *p* < 0.001), but not the covariate. On **(A–C)** *p*-value corrected for multiple comparisons to < 0.01. ^*^p < 0.05, ^**^p < 0.01, ^***^p < 0.001. DERS, Difficulties in Emotion Regulation Scale; BPD, borderline personality disorder; CD, Cocaine dependence.

For the DERS (emotional regulation), the total score did not differ between BPD+CD+ and BPD+CD− groups (BPD+CD+ M = 70.61, BPD+CD− M = 77.67, *U* = 62.0, *p* = 0.348). As indicated in Figure 2, the differences between BPD+CD+ and BPD−CD+, as well as between the BPD-CD+ and BPD+CD− groups approached significance. The ANOVA could be performed only for the non-acceptance and goals subscales, after normalizing through a square root transformation. For non-acceptance, only the BPD factor was significant (*F* = 34.7, *p* < 0.001) with an *R*^2^ of 0.407. With current dysthymia as covariate the *R*^2^ increases to 0.442 and with cigarettes/day, it diminishes to 0.368 with a nearly significant interaction (*F* = 3.78, *p* = 0.057), and BPD remaining the only significant effect (*F* = 24.31*, p* < 0.001). As shown in Figure 2, a negative interaction between BPD and CD factors was found with the goals subscale, meaning counteracting effects of the factors.

### Neuroimaging findings

**Figure 3:**
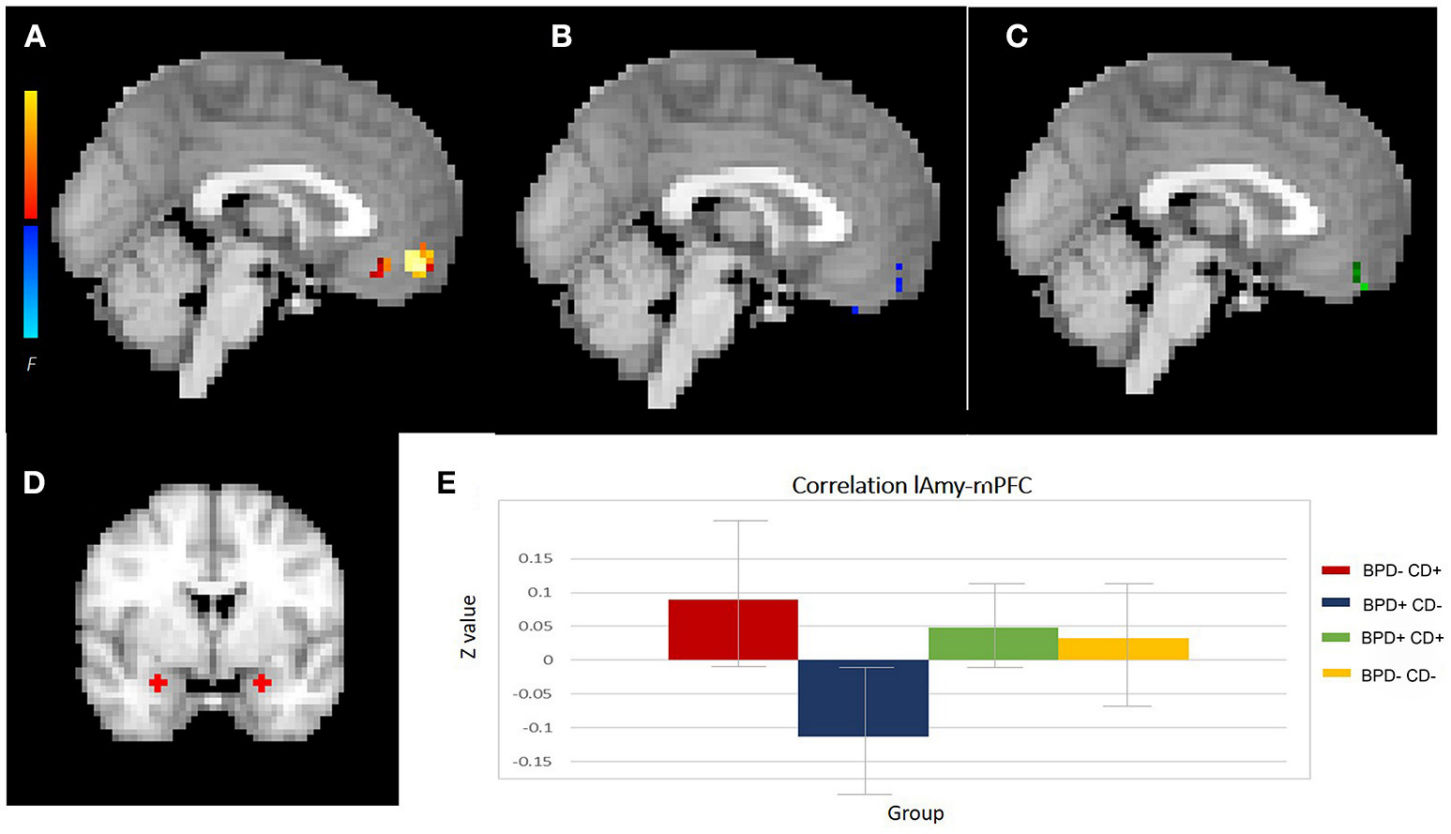
Amygdala connectivity. **(A–C**) Show the significant clusters from lAmy-mPFC connectivity analysis: **(A)** the CD effect, **(B)** the BPD effect and **(C)** the interaction. **(D)** Left and right amygdala seeds. **(E)** resting state functional connectivity effect sizes for each group with 95% confidence intervals (error bars). lAmy, left amygdala; mPFC, medial prefrontal cortex; CD, cocaine dependence; BPD, borderline personality disorder.

**Figure 4:**
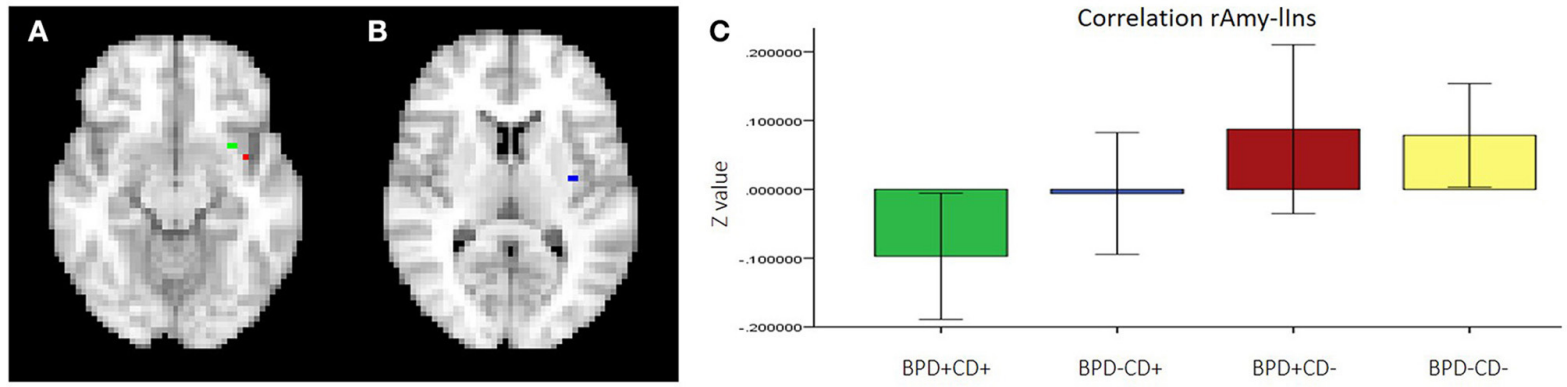
Functional connectivity between rAmy-lIns. **(A)** Clusters 1 (green) and 3 (red), **(B)** Cluster 2, **(C)** media correlation of each group with a confidence interval at 95%. Note: rAmy, right amygdala; lIns, left insula; BPD, borderline personality disorder; CD, cocaine dependence.

For the neuroimaging analysis, two participants from the BPD-CD- group were excluded due to poor image quality, leaving *n* = 67 for analysis. The two-way ANOVA analysis of functional connectivity, controlled for confounds variables: sex, age, education, current major depressive disorder, current dysthymia and current alcohol use, showed a significant interaction effect between BPD and CD factors in lAmy and mPFC connectivity. This was a negative interaction between the factors, meaning a counteracting effect of each factor: while the BPD-CD+ group had an increased connectivity (significant main effect), BPD+CD− had a decreased connectivity (not significant main effect). The connectivity of the BPD+CD+ group was similar to the BPD−CD− group. We also found a significant interaction effect between rAmy and lIns connectivity, whereby having both factors reduced the connectivity. Details are shown in Figures 3, 4 and Table 2. The analysis of DMN connectivity showed no significant effects.

**Table 2:** Significant clusters from amygdala connectivity analyses.

**Connectivity analysis factor**	**Area**	**Cluster size (voxels)**	**Maximum**			
				**MNI coordinates**		
			**Significance (*p)***	***X***	***Y***	***Z***
**lAmy—mPFC**						
Interaction	Right frontopolar cortex	6	0.032	−3	48	−21
Cocaine	Right frontopolar cortex	42	0.008	0	51	−15
Borderline personality disorder	Right frontopolar cortex	1	0.055	−3	51	−9
**rAmy-lIns**						
Interaction						
Cluster 1	Left insula	3	0.015	−30	9	−12
Cluster 2	Left insula	2	0.037	−36	−9	9
Cluster 3	Left insula	1	0.049	−39	−12	−12

**Figure 5:**
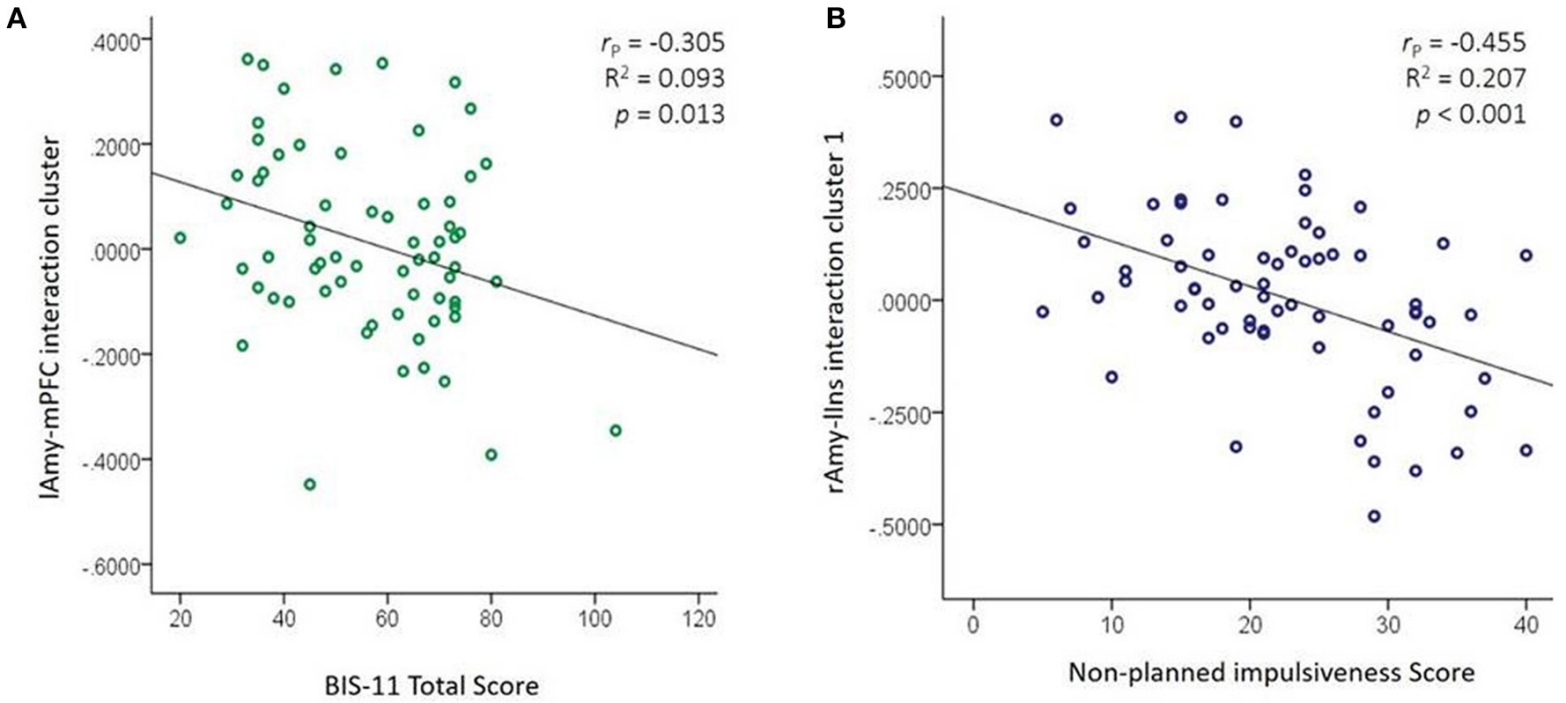
Associations between amygdala functional connectivity and impulsivity. **(A)** lAmy-mPFC connectivity from cluster resulted from the BPD × CD interaction with total BIS-11 score correlation **(B)** rAmy-lIns connectivity from cluster 1 resulted from the BPD × CD interaction with non-planned BIS-11 score correlation. l/rAmy, left/right amygdala; mPFC, medial prefrontal cortex; BPD, borderline personality disorder; CD, cocaine dependence; BIS-11, Barratt Impulsiveness Scale.

The mean correlations obtained from the significant clusters were correlated with BIS-11 and DERS total and subscales scores. We obtained 12 significant correlations shown in Table 3. The strongest correlation was a negative correlation between the cluster from the interaction effect of lAmy-mPFC connectivity with BIS-11 total score. That is, stronger connectivity between lAmy and mPFC was related to lower scores on self-reported impulsivity. Another negative correlation found was between the first cluster from the interaction effect at rAmy-lIns and non-planning impulsivity. That is, higher rAmy-lIns functional connectivity was related to lower non-planning impulsivity. These two correlations are shown in Figure 5.

**Table 3:** Associations between significant clusters functional connectivity and clinical measures.

**Cluster Clinical measure**	**lAmy-mPFC interaction**	**lAmy-mPFC BPD**	**rAmy-lIns interaction cluster 1**
BIS total	**−0.305 (0.013)**	**−0.255 (0.039)**	**−0.286 (0.020)**
BIS cognitive	−**0.288 (0.019)**	−0.238 (0.054)	−0.070 (0.575)
BIS motor	−0.217 (0.081)	−0.206 (0.097)	−0.079 (0.530)
BIS non–planned	−0.239 (0.053)	−0.180 (0.149)	**−0.455 (<0.001)**
DERS total	−0.135 (0.291)	−0.075 (0.558)	**−0.277 (0.028)**
DERS non–acceptance	**−0.278 (0.023)**	**−0.268 (0.028)**	−0.210 (0.088)
DERS goals	**−0.246 (0.045)**	**−0.274 (0.025)**	−0.181 (0.143)
DERS awareness	−0.146 (0.238)	−0.169 (0.172)	**−0.271 (0.026)**
DERS clarity	−0.**248** (0.043)	−0.213 (0.084)	−0.238 (0.052)

*lAmy-mPFC CD, rAmy-lIns interaction clusters 2 and 3 did not presented any significant correlation*.

*Numbers represents: Pearson coefficient (p value)*.

*BIS, Barratt Impulsiveness Scale; DERS, Difficulties in Emotion Regulation Scale; l/r Amy, left/right amygdala; mPFC, medial Prefrontal Cortex; lIns, left Insula; BPD, Borderline Personality Disorder*.

## Discussion

We sought to understand the psychopathology of borderline personality disorder (BPD) with cocaine dependency (CD), using clinical and functional connectivity measures. We found that the dual pathology group (BPD+CD+) resembled the only borderline personality disorder group (BPD+CD−) in impulsivity and emotional dysregulation scores more than the only cocaine dependence group (BPD−CD+). We also found that patients with BPD+CD+ displayed a similar lAmy-mPFC functional connectivity pattern to the healthy control group (BPD−CD−, while the rAmy—lIns functional connectivity pattern was opposite to the healthy control group. In addition, self-reported impulsivity correlated negatively with significant clusters from amygdala functional connectivity. To our knowledge, this is the first study to investigate the effect of this dual pathology using clinical and neuroimaging methods.

### Psychiatric comorbidities, impulsivity and emotional dysregulation

We found greater prevalence of current major depressive episode and dysthymia in the BPD+CD− group. This could reflect the greater proportion of women in that group, as mood disorders are more frequent among women than men with BPD (41–44). We also found higher current alcohol consumption in the BPD−CD+ group, which was expected as the presence of a substance use disorder increases the likelihood of abusing other substances, especially for males (1, 42); our BPD−CD+ group had the highest proportion of males. The number of cigarettes consumed per day was highest in the BPD+CD+ group. Nicotine consumption has been associated with use of other substances and with BPD (45). Introducing these variables in the remaining analyses can reduce their influence in the inference models, as they are difficult to control in composition of the groups due to the nature of the study population.

Impulsivity and emotional dysregulation are core characteristics of BPD. Impulsivity was described as the main predictor of borderline symptomatology in a seven year follow-up (14), while emotional dysregulation predicts aggressive behavior even more than impulsivity (46), and is a process that is impaired in patients with BPD (47) and in CD (16). With respect to CD, impulsivity predicts higher cocaine consumption (13) and increases the risk of stimulant use disorder (12). Also, during brief periods of abstinence, patients with CD show emotional dysregulation (16). In previous studies of dual pathology (BPD and CD), the dual pathology group presented more severe difficulties with sexual risk behaviors (8) and greater attentional bias toward cocaine cues under an emotional stress condition (9) than groups with only one of the disorders. Taking this into account, clinically we expected an additive effect of BPD combined with CD on impulsivity and emotional dysregulation. However, we found similar scores in both constructs for patients with BPD+CD+ and BPD+CD−, and a significant effect of the interaction for the total BIS-11 score, that was maintained after adding the confounding variables to the model, except antidepressant use and daily cigarette consumption. The loss of significant interaction when adding these last two variables may be due to a statistical power reduction or from an effect of those psychoactive substances, although they do not present a significant effect by themselves according to our analyses. A study without antidepressant and tobacco may prove challenging as our patients must be in treatment and cocaine addicts usually smoke tobacco cigarettes.

### Functional connectivity

We found a significant interaction in the functional connectivity of left amygdala—mPFC in the BPD and CD factors of the ANCOVA, showing opposite effects (BPD showed higher connectivity while CD showed lower connectivity) and thus reducing the functional connectivity in the BPD+CD+ group down to a similar level as the BPD−CD− group (healthy controls). The functional connectivity strength of the lAmy—mPFC circuit was negatively correlated with impulsivity and emotional dysregulation, meaning that the connectivity was lower when these clinical measures where higher. These findings make sense in light of what has been described in literature about amygdala—mPFC functional connectivity related to emotion regulation and impulsivity, as well as our clinical findings. This is clearly observed in the BPD+CD− group, which resulted with the lowest connectivity and highest impulsivity and emotional dysregulation scores. On the other hand, the BPD+CD+ group did not show a low functional connectivity even with the high impulsivity and emotional dysregulation, as the BPD+CD− group did. This may be the effect of presenting a dual pathology and the possible use of cocaine as means of empirical self-regulation. The amygdala is involved in processing emotional stimuli, while the mPFC is involved in regulating emotional conflict and modulating emotional responses through reappraisal. Studies have shown that amygdala—mPFC connectivity is important for top-down emotion regulation (48, 49), and the strength of the connectivity seems to be positively correlated with the effectiveness of emotional regulation in a reappraisal task (50). In substance dependence it has also been suggested to facilitate the urgency to consume during abstinence, signaling to a so-called “impulsive system” (consisting of the amygdala and nucleus accumbens), that magnifies the value of somatic marker representations (51). Moreover, it has been reported that amygdala functional activations are lateralized to the left in emotional task-fMRI studies (52). In the BPD+CD− group, our results agree with previous studies that did not find differences in amygdala—mPFC functional connectivity compared with BPD−CD− (53, 54). In the BPD−CD+ group we did not find a significant effect on functional connectivity, although it has been reported to be lower in CD compared to controls (20). However, this may be due to our sample size. The sample in their study was older and without any psychiatric comorbidity, and their resting state fMRI preprocessing also differed from ours, which may account for the discrepancy.

Another relevant finding was the significant interaction effect in the functional connectivity of rAmy—lIns, where having dual pathology significantly diminished their connectivity, in an additive manner. The functional connectivity was negatively correlated with emotional dysregulation and impulsivity scores, especially with non-planned impulsivity, which relates to self-control and cognitive complexity (55). The insula has been linked to emotion regulation (56) and impulsivity (57, 58), and is a key component of the salience network which is activated in sensory stimulus-guided goal-directed behaviors (59). In BPD patients, insula function has been related to emotional processing of pain (60). In substance use disorders, craving involves a dysregulation of afferent projections from the insula to amygdala and related structures (61). Our finding about the additive effects of both factors (BPD and CD) in rAmy-lIns connectivity in dual pathology patients, as well as the negative correlations with impulsivity and emotional regulation, may help to explain the greater attentional bias to cocaine-related visual stimuli under emotional stress in the dual pathology group found in another study (9). It has been proposed that the amygdala is involved with fast, short and relatively automatic processes (62) such as the detection of salient stimuli. Under this model, aberrant rAmy–lIns functional connectivity would impair emotion regulation under stress and make cocaine cues more salient.

The similar impulsivity and functional connectivity patterns found between dual pathology patients and healthy controls could be explained as possible counteracting effects of each disorder. Our findings suggest BPD may be the main pathology of this dual pathology, and the cocaine consumption may act as a compensatory behavior to self-regulate emotion and impulsivity, until it becomes substance dependency. The similar functional connectivity pattern between dual pathology and healthy controls has been described in patients with schizophrenia and cannabis dependency, where the cannabinoid administration improved DMN connectivity (63). The connectivity differences between groups in amygdala-insula connectivity may be related to the substance dependence or a predisposing trait to become dependent in this population, though this needs to be confirmed in longitudinal studies. Overall, our clinical and functional connectivity results are related to each other, and we suggest that cocaine dependence in this case may be an empirical method of self-regulating the amygdala—mPFC connectivity in BPD that, at the same time, may negatively affect other circuits such as amygdala—insula connectivity, and therefore, enhancing attentional bias to cocaine cues in dual pathology patients.

Clinical guidelines and the literature indicate that when a dual pathology diagnosis is present, both disorders must be treated in an integrated manner^2^ (64). However, mental health teams able to manage dual diagnosis patients remain scarce and currently, the common clinical practice is to treat the substance use disorder predominantly or exclusively, even in specialized clinics (65). In our study we suggest that BPD may be predominant in dual pathology, and CD may be a consequence of the psychopathology. If this is the case, by treating the BPD symptoms the CD should be easier to manage, and therefore, we suggest future clinical trials should address this hypothesis.

### Limitations

The main limitation of our study is the small sample size of groups, especially the BPD+CD− group, which limited our statistical power. When we introduced the clinical variables that were different between the clinical groups in the analysis (current major depressive disorder, current dysthymia, current alcohol use, number of cigarettes consumed per day and antidepressant use), a number of significant results changed, making it difficult to assess their impact. Despite this, our sample was uniform on illnesses severity and on most clinical variables. In neuroimaging, sample sizes of at least 22 subjects per group are recommended for task-based studies (66). No such estimates are available for resting state studies and reaching those sample sizes would have been extremely difficult given the types of patients we studied. Therefore, we limited our analyses to candidate-circuits based on prior evidence, greatly reducing our multiple-comparisons problem.

Another issue is that most CD studies have included only males, and BPD studies have tended to include only females, while we included both sexes in most our groups. We decided to include patients from both sexes because dual pathology affects both, male and female patients. As mentioned in the introduction, from previous clinical studies (8, 9) there seems to be an effect of sex in the presentation of this dual pathology. We added sex as a control variable in the analyses, but our sample size does not allow sex as a factor to analyze. There is a need for studies with a larger sample sizes that could address this part of the dual disorder. A further limitation is that our participants were recruited from a clinical population seeking treatment in specialized units, making it difficult to generalize the results to the broader population of patients not in treatment. Although some of our patients were in medication and that could affect our results, only antidepressant use showed differences between groups and it was added as a confounding variable. We used only self-reported measures for emotional dysregulation and impulsivity which may be a bias. However, these measures are part of the research field and a trained psychiatrist who knew the patients reviewed their responses to corroborate. Despite these limitations, this is the first study that examines impulsivity, emotional dysregulation and functional connectivity in this dual pathology (borderline personality disorder and cocaine dependence).

## Conclusions

In summary, we found that patients with dual pathology showed similar difficulties in impulsivity and emotional dysregulation as those only borderline personality disorder, and they had similar resting state amygdala—mPFC functional connectivity as healthy controls. Also, the dual pathology group showed reduced amygdala—insula functional connectivity compared to the other groups. This suggests the speculative hypothesis that cocaine consumption may be a form of self-medication in borderline personality disorder to normalize amygdala—mPFC connectivity.

## Ethics statement

This study was carried out in accordance with the recommendations of the Ethics Committee of the National Institute of Psychiatry Ramón de la Fuente Muñiz. The protocol was approved by the Ethics Committee of the National Institute of Psychiatry Ramón de la Fuente Muñiz. All subjects gave written informed consent in accordance with the Declaration of Helsinki.

## Author contributions

TB, EG-V, and JG-O were involved in the design of the research protocol. TB, EG-V, DA-V, and IE-L contributed in acquisition and analysis of data. TB and EG-V drafted the manuscript and all authors contributed revising and approved it for publication.

### Conflict of interest statement

The authors declare that the research was conducted in the absence of any commercial or financial relationships that could be construed as a potential conflict of interest.

## Footnotes

^1^United Nations Office on Drugs and Crime; World drug report 2009. 2009. [cited 2018 May 8] Available from: https://www.unodc.org/documents/wdr/WDR_2009/WDR2009_eng_web.pdf

^2^Department of Health. Mental Health Policy Implementation Guide: Dual Diagnosis Good Practice Guide 2002 [Internet]. 2002. Available from: http://webarchive.nationalarchives.gov.uk/20121012200956/http://www.dh.gov.uk/prod_consum_dh/groups/dh_digitalassets/@dh/@en/documents/digitalasset/dh_4060435.pdf

## Abbreviations

ACC: Anterior cingulate cortex
Amy (rAmy/lAmy): Amygdala (right amygdala/left amygdala)
BIS-11: Barratt Impulsiveness Scale
BPD: Borderline personality disorder
CD: Cocaine dependence
DERS: Difficulties in Emotion Regulation Scale
DMN: Default mode network
fMRI: Functional magnetic resonance imaging
rIns/lIns: Right insula/left insula
MNI: Montreal Neurological Institute (stereotactic space)
mPFC: Medial prefrontal cortex
PCC: Posterior cingulate cortex.
