# Supplementary material for: Borderline Personality Disorder With Cocaine Dependence: Impulsivity, Emotional Dysregulation and Amygdala Functional Connectivity
Source: Front Psychiatry. 2018 Jul 31;9:328. doi: 10.3389/fpsyt.2018.00328 (PMC6079279; doi:10.3389/fpsyt.2018.00328)
Supplement: Supplementary file 1 [file Table_1.DOCX]

Supplementary Material

**Borderline personality disorder with cocaine dependence: impulsivity, emotional dysregulation and amygdala functional connectivity**

**Thania Balducci, Jorge J González-Olvera, Diego Angeles-Valdez, Isabel Espinoza-Luna, Eduardo A Garza-Villarreal^*^**

*** Correspondence:** Eduardo A Garza-Villarreal: egarza@imp.edu.mx

# Supplementary Table 1S

| **Tabla 1S**. Psychiatric comorbidity, substance use and medication | | | | | |
| --- | --- | --- | --- | --- | --- |
|  | BPD+CD+  (n = 20) | BPD-CD+  (n = 19) | BPD+CD-  (n = 10) | *X*^2^ value | *p value* |
| No. of psychiatric comorbidities, median (rank)  current  past^†^ | 1 (0-4)  1 (0-2) | 1 (0-3)  0 (0-2) | 1 (0-3)  0 (0-3) | 2.103  0.946 | 0.349  0.623 |
| Disorders, n (%)  Current MDE  Past MDE  Current dysthymia  Past dysthymia  Mania episode secondary to amphetamines  Current panic disorder  Past panic disorder  Panic disorder secondary to substances  Social phobia  Specific phobia  Current PTSD  Generalized anxiety disorder  Other anxiety disorder secondary to substances  ADHD on childhood  ADHD on adulthood | -  3 (15.0)  2 (10.0)  -  -*  1 (5.3)*  -*  -  -  3 (15.0)  2 (10.5)*  5 (26.3)*  1 (5.0)  11 (55.0)  7 (35.0) | 2 (10.5)  4 (21.1)  -  -  1 (5.3)  -  1 (5.3)  1 (5.3)  1 (5.3)  3 (15.8)  -  1 (5.6)*  -  7 (38.9)*  4 (21.1)* | 3 (30.0)  3 (30.0)  3 (30.0)  1 (10.0)  -  -  -  NA  -  -  -  3 (30)  NA  1 (10.0)  - | 6.552  0.931  6.437  3.981  1.559  1.559  1.559  1.612  1.612  1.579  1.759  3.185  1.612  5.651  5.390 | < 0.05  0.628  < 0.05  0.137  0.459  0.459  0.459  0.447  0.447  0.454  0.203  0.170  0.447  0.059  0.068 |
| Number of substances used, median (rank)  Total  Current  Past  Experimental  Substance, n (%)  Current alcohol  Past alcohol  Current hallucinogen  Experimental hallucinogen  Current inhalants  Past inhalants  Experimental inhalants  Current cannabis  Past cannabis  Experimental cannabis  Current benzodiazepines  Past benzodiazepines  Past steroids  Nicotine cigarette/day, media (SD)^‡^  Drug group, n (%)  Anticonvulsants  Neuroleptics  Benzodiazepines  Antidepresants  Unknown | 4 (3-6)  2 (1-4)  2 (0-3)  0 (0-4)  6 (30.0)  5 (25.0)  -  4 (20.0)  2 (10.0)  1 (5.0)  4 (20.0)  6 (30.0)  1 (5.0)  6 (30.0)  1 (5.0)  -  1 (5.0)  7.65 (8.44)  6 (30.0)  1 (5.0)  2 (10.0)  3 (15.0)  - | 3 (1-6)  1 (1-4)  0 (0-3)  0 (0-2)  13 (72.2)*  1 (5.6)*  1 (5.6)*  2 (11.1)*  3 (16.7)*  -  1 (5.6)*  4 (22.2)*  1 (5.6)*  6 (33.3)*  -  1 (5.6)*  -  1.63 (1.63)  5 (26.3)  1 (5.3)  -  -  2 (10.5) | 0.5 (0-3)  1 (0-3)  0 (0-2)  0 (0-1)  2 (20)  -  -  -  -  -  -  1 (10.0)  2 (20.0)  -  -  -  -  0.70 (2.21)  1 (10.0)  3 (30.0)  -  7 (70.0)  - | 15.16  4.266  1.655  5.411  9.758  5.079  1.702  2.489  1.920  1.670  3.587  1.517  2.255  4.267  1.430  1.702  1.430  20.521  1.498  5.374  3.023  20.37  - | < 0.001  0.118  0.437  0.067  < 0.01  0.079  0.427  0.288  0.383  0.434  0.166  0.468  0.324  0.118  0.489  0.427  0.489  < 0.001  0.473  0.068  0.221  < 0.001  - |
| Note: p value obtained through x2 for categorical variables and Kruskal-Wallis for ordinal variables and for cigarettes/day, being this the only one-dimensional variable with unequal variance (Levene 12.49, p < 0.001). On substance use, we do not distinguish between dependence, abuse or another pattern of consume. At the BPD group, none had a pattern of consume larger than social on any substance but nicotine.  * One value lost (n-1).  ^†^ Past comorbidities: past MDE, past dysthymia, past panic disorder, ADHD on childhood.  ^‡^ On this analysis, the control group without psychopathology was also included with a media of consumed cigarettes/day of 0.95 and SD 1.35.  BPD: borderline personality disorder, CD: cocaine dependence | | | | | |
