# Supplementary material for: Borderline Personality Disorder With Cocaine Dependence: Impulsivity, Emotional Dysregulation and Amygdala Functional Connectivity
Source: Front Psychiatry. 2018 Jul 31;9:328. doi: 10.3389/fpsyt.2018.00328 (PMC6079279; doi:10.3389/fpsyt.2018.00328)
Supplement: Supplementary file 2 [file Table_2.DOCX]

Supplementary Material

**Borderline personality disorder with cocaine dependence: impulsivity, emotional dysregulation and amygdala functional connectivity**

**Thania Balducci, Jorge J González-Olvera, Diego Angeles-Valdez, Isabel Espinoza-Luna, Eduardo A Garza-Villarreal^*^**

*** Correspondence:** Eduardo A Garza-Villarreal: egarza@imp.edu.mx

# Supplementary Table 2S

| **Table 2S**. ANOVA and ANCOVA results for BIS-11 | | | | | | |
| --- | --- | --- | --- | --- | --- | --- |
| Model  Source | Mean (DE) | Type III Sum of squares | df | F | Sig | R^2^ adjusted |
| BIS total |  |  |  |  |  | 0.325 |
| BPD | 62.32 (14.26) | 4310.7 | 1 | 20.75 | < 0.001 |  |
| CD | 47.87 (16.76) | 1275.4 | 1 | 6.14 | 0.016 |  |
| BPD x CD |  | 893.1 | 1 | 4.29 | 0.042 |  |
| Error |  | 1391.0 | 67 |  |  |  |
| BIS total, CMDE |  |  |  |  |  | 0.413 |
| CMDE |  | 1.0 | 1 | 1.54 | 0.220 |  |
| BPD |  | 15.9 | 1 | 23.84 | < 0.001 |  |
| CD |  | 7.0 | 1 | 10.48 | 0.002 |  |
| BPD x CD |  | 2.1 | 1 | 3.11 | 0.083 |  |
| Error |  | 40.6 | 61 |  |  |  |
| BIS total, CDys |  |  |  |  |  | 0.435 |
| CDys |  | 2.5 | 1 | 3.89 | 0.053 |  |
| BPD |  | 12.7 | 1 | 19.88 | < 0.001 |  |
| CD |  | 7.7 | 1 | 11.97 | 0.001 |  |
| BPD x CD |  | 3.1 | 1 | 4.80 | 0.032 |  |
| Error |  |  | 61 |  |  |  |
| BIS total, CAlc |  |  |  |  |  | 0.398 |
| CAlc |  | 0.0 | 1 | 0.00 | 0.985 |  |
| BPD |  | 15.5 | 1 | 22.45 | < 0.001 |  |
| CD |  | 5.9 | 1 | 8.52 | 0.005 |  |
| BPD x CD |  | 3.7 | 1 | 5.32 | 0.024 |  |
| Error |  | 41.5 | 60 |  |  |  |
| BIS total, AD |  |  |  |  |  | 0.422 |
| AD |  | 0.3 | 1 | 0.35 | 0.555 |  |
| BPD |  | 11.3 | 1 | 14.60 | < 0.001 |  |
| CD |  | 8.1 | 1 | 10.48 | 0.002 |  |
| BPD x CD |  | 2.3 | 1 | 2.92 | 0.092 |  |
| Error |  | 3804.0 | 63 |  |  |  |
| BIS total, Cigarettes |  |  |  |  |  | 0.489 |
| Cigarettes |  | 0.1 | 1 | 0.21 | 0.644 |  |
| BPD |  | 24.4 | 1 | 24.43 | < 0.001 |  |
| CD |  | 8.8 | 1 | 8.76 | 0.001 |  |
| BPD x CD |  | 1.2 | 1 | 1.23 | 0.191 |  |
| Error |  | 39.5 | 56 |  |  |  |
| BIS cognitive |  |  |  |  |  | 0.313 |
| BPD | 16.89 (4.09) | 273.5 | 1 | 19.23 | < 0.001 |  |
| CD | 14.00 (4.65) | 51.7 | 1 | 3.64 | 0.61 |  |
| BPD x CD |  | 92.4 | 1 | 6.50 | 0.13 |  |
| Error |  | 867.6 | 61 |  |  |  |
| BIS cognitive, CMDE |  |  |  |  |  | 0.293 |
| CMDE |  | 0.2 | 1 | 0.01 | 0.912 |  |
| BPD |  | 389.1 | 1 | 20.10 | < 0.001 |  |
| CD |  | 69.5 | 1 | 3.59 | 0.063 |  |
| BPD x CD |  | 53.7 | 1 | 2.78 | 0.101 |  |
| Error |  | 1219.3 | 63 |  |  |  |
| BIS cognitive, CDys |  |  |  |  |  | 0.311 |
| CDys |  | 32.8 | 1 | 1.74 | 0.192 |  |
| BPD |  | 305.9 | 1 | 16.24 | < 0.001 |  |
| CD |  | 89.0 | 1 | 4.73 | 0.033 |  |
| BPD x CD |  | 53.3 | 1 | 2.83 | 0.097 |  |
| Error |  | 1186.7 | 63 |  |  |  |
| BIS cognitive, CAlc |  |  |  |  |  | 0.308 |
| CAlc |  | 9.9 | 1 | 0.53 | 0.470 |  |
| BPD |  | 420.4 | 1 | 22.39 | < 0.001 |  |
| CD |  | 50.2 | 1 | 2.67 | 0.107 |  |
| BPD x CD |  | 52.5 | 1 | 2.79 | 0.099 |  |
| Error |  | 1163.9 | 62 |  |  |  |
| BIS cognitive, AD |  |  |  |  |  | 0.337 |
| AD |  | 76.2 | 1 | 4.20 | 0.045 |  |
| BPD |  | 108.9 | 1 | 6.00 | 0.017 |  |
| CD |  | 130.3 | 1 | 7.18 | 0.009 |  |
| BPD x CD |  | 11.3 | 1 | 0.62 | 0.433 |  |
| Error |  | 1143.3 | 63 |  |  |  |
| BIS cognitive, Cigarettes |  |  |  |  |  | 0.414 |
| Cigarettes |  | 104.3 | 1 | 6.30 | 0.015 |  |
| BPD |  | 597.0 | 1 | 36.08 | < 0.001 |  |
| CD |  | 123.5 | 1 | 7.46 | 0.008 |  |
| BPD x CD |  | 1.5 | 1 | 0.91 | 0.764 |  |
| Error |  | 926.7 | 56 |  |  |  |
| BIS motor |  |  |  |  |  | 0.305 |
| BPD | 19.42 (7.21) | 1054.7 | 1 | 26.64 | < 0.001 |  |
| CD | 16.10 (7.68) | 32.8 | 1 | 0.83 | 0.366 |  |
| BPD x CD |  | 145.4 | 1 | 3.67 | 0.060 |  |
| Error |  | 2533.5 | 64 |  |  |  |
| BIS motor, CMDE |  |  |  |  |  | 0.294 |
| CMDE |  | 1.6 | 1 | 0.040 | 0.841 |  |
| BPD |  | 1000.3 | 1 | 24.89 | < 0.001 |  |
| CD |  | 34.4 | 1 | 0.85 | 0.359 |  |
| BPD x CD |  | 114.2 | 1 | 2.84 | 0.097 |  |
| Error |  | 2531.8 | 63 |  |  |  |
| BIS motor, CDys |  |  |  |  |  | 0.314 |
| CDys |  | 73.4 | 1 | 1.88 | 0.175 |  |
| BPD |  | 802.3 | 1 | 20.55 | < 0.001 |  |
| CD |  | 55.2 | 1 | 1.41 | 0.239 |  |
| BPD x CD |  | 117.7 | 1 | 3.01 | 0.087 |  |
| Error |  | 2460.0 | 63 |  |  |  |
| BIS motor, CAlc |  |  |  |  |  | 0.300 |
| CAlc |  | 25.2 | 1 | 0.624 | 0.432 |  |
| BPD |  | 998.1 | 1 | 24.75 | < 0.001 |  |
| CD |  | 26.9 | 1 | 0.67 | 0.417 |  |
| BPD x CD |  | 147.6 | 1 | 3.66 | 0.060 |  |
| Error |  | 2499.8 | 62 |  |  |  |
| BIS motor, AD |  |  |  |  |  | 0.301 |
| AD |  | 25.2 | 1 | 0.63 | 0.429 |  |
| BPD |  | 500.8 | 1 | 12.58 | 0.001 |  |
| CD |  | 54.2 | 1 | 1.36 | 0.248 |  |
| BPD x CD |  | 73.0 | 1 | 1.83 | 0.180 |  |
| Error |  | 2508.3 | 63 |  |  |  |
| BIS motor, Cigarettes |  |  |  |  |  | 0.361 |
| Cigarettes |  | 12.3 | 1 | 0.34 | 0.560 |  |
| BPD |  | 1096.2 | 1 | 30.67 | < 0.001 |  |
| CD |  | 65.1 | 1 | 1.82 | 0.183 |  |
| BPD x CD |  | 41.2 | 1 | 1.15 | 0.288 |  |
| Error |  | 2001.6 | 56 |  |  |  |
| BIS non-planed |  |  |  |  |  | 0.210 |
| BPD | 18.48 (8.13) | 212.5 | 1 | 4.09 | 0.047 |  |
| CD | 25.57 (6.67) | 599.9 | 1 | 11.56 | 0.001 |  |
| BPD x CD |  | 46.6 | 1 | 0.96 | 0.332 |  |
| Error |  | 3218.4 | 62 |  |  |  |
| BIS non-planed, CMDE |  |  |  |  |  | 0.268 |
| CMDE |  | 166.3 | 1 | 3.18 | 0.079 |  |
| BPD |  | 201.5 | 1 | 3.85 | 0.054 |  |
| CD |  | 839.8 | 1 | 16.06 | < 0.001 |  |
| BPD x CD |  | 1.5 | 1 | 0.03 | 0.866 |  |
| Error |  | 3293.2 | 63 |  |  |  |
| BIS, CDys |  |  |  |  |  | 0.244 |
| CDys |  | 59.9 | 1 | 1.11 | 0.296 |  |
| BPD |  | 191.1 | 1 | 3.54 | 0.064 |  |
| CD |  | 786.2 | 1 | 14.57 | < 0.001 |  |
| BPD x CD |  | 30.4 | 1 | 0.56 | 0.456 |  |
| Error |  | 3399.7 | 63 |  |  |  |
| BIS non-planed, CAlc |  |  |  |  |  | 0.247 |
| CAlc |  | 76.8 | 1 | 1.41 | 0.240 |  |
| BPD |  | 154.6 | 1 | 2.83 | 0.097 |  |
| CD |  | 779.8 | 1 | 14.29 | < 0.001 |  |
| BPD x CD |  | 46.9 | 1 | 0.86 | 0.357 |  |
| Error |  | 3382.3 | 62 |  |  |  |
| BIS non-planed, AD |  |  |  |  |  | 0.239 |
| AD |  | 33.7 | 1 | 0.62 | 0.434 |  |
| BPD |  | 286.6 | 1 | 5.27 | 0.025 |  |
| CD |  | 465.5 | 1 | 8.56 | 0.005 |  |
| BPD x CD |  | 71.7 | 1 | 1.32 | 0.255 |  |
| Error |  | 3425.8 | 63 |  |  |  |
| BIS non-planed, Cigarettes |  |  |  |  |  | 0.264 |
| Cigarettes |  | 62.8 | 1 | 1.10 | 0.298 |  |
| BPD |  | 239.4 | 1 | 4.21 | 0.045 |  |
| CD |  | 516.4 | 1 | 9.09 | 0.004 |  |
| BPD x CD |  | 49.4 | 1 | 0.87 | 0.355 |  |
| Error |  | 3182.8 | 56 |  |  |  |

Note: Covariables used for the analysis: CMDE Current major depressive episode CDys Current Dysthymia CAlc Current alcohol use disorder AD antidepressants Cigarettes/day. BIS total score normalized by square root. Significant p < 0.05

BIS-11: Barratt Impulsiveness Scale, BPD: borderline personality disorder, CD: cocaine dependence
